# EFFECTS OF A SHARED DECISION MAKING INTERVENTION FOR OLDER ADULTS WITH CHRONIC CONDITIONS: AN OBSERVATIONAL STUDY

**DOI:** 10.1093/geroni/igz038.1090

**Published:** 2019-11-08

**Authors:** Ruth E Pel-Littel, Bianca Buurman, Marjolein van de Pol, Linda Tulner, Mirella Minkman, Wilma Scholte op Reimer, Julia van Weert

**Affiliations:** 1 Vilans, Utrecht, Utrecht, Netherlands; 2 Department of Internal Medicine, Section of Geriatric Medicine, Academic Medical Centre, University of Amsterdam, Amsterdam, Netherlands; 3 Department of Primary and Community Care, Radboud University Medical Center, Nijmegen, Netherlands; 4 Medisch Centrum Slotervaart, Amsterdam, Netherlands; 5 Vilans, Utrecht, Netherlands; 6 ACHIEVE, Centre of Applied Research, Faculty of Health, Amsterdam University of Applied Sciences, Amsterdam, Netherlands; 7 Amsterdam School of Communication Research/ASCoR, University of Amsterdam, Amsterdam, Netherlands

## Abstract

Shared decision making (SDM) in older patients is more complex when multiple chronic conditions (MCC) have to be taken into account. The aim of this research is to explore the effect of the evidence based implementation intervention SDMMCC on (1) the preferred and perceived participation (2) decisional conflict and (3) actual SDM during consultations. 216 outpatients participated in a video observational study. The intervention existed of a SDM training for geriatricians and a preparatory tool for patients. Consultations were videotaped and coded with the OPTIONMCC. Pre- and post-consultation questionnaires were completed. Participation was measured by the Patients’ perceived Involvement in Care Scale (PICS). Decisional conflict was measured by the Decisional Conflict Scale (DCS). The patients mean age was 77 years, 56% was female. The preparatory tool was completed by 56 older adults (52%), of which 64% rated the tool as positive. The preparatory tool was used in 12% of the consultations. The mean overall OPTIONMCC score showed no significant changes on the level of SDM(39.3 vs 39.3 P0.98), however there were significant improvements on discussing goals and options on sub-items of the scale. There were no significant differences found in the match on preferred and perceived participation (86.5% vs 85.0% P 0.595) or in decisional conflict (22.7 vs 22.9 P0.630). The limited use of the preparatory tool could have biased the effect of the intervention. In future research more attention must be paid towards the implementation of preparatory tools, not only among patients but also among geriatricians.

